# In Vitro Efficacy and Toxicity Assessment of an Amphotericin B Gel for the Treatment of Cutaneous Leishmaniasis

**DOI:** 10.3390/ph18030427

**Published:** 2025-03-18

**Authors:** Lilian Sosa, Lupe Carolina Espinoza, Marcelle Silva-Abreu, Ximena Jaramillo-Fierro, Diana Berenguer, Cristina Riera, María Rincón, Ana C. Calpena

**Affiliations:** 1 Microbiology Research Institute (IIM), Faculty of Sciences, National Autonomous University of Honduras (UNAH), Tegucigalpa 11101, Honduras; lilian.sosa@unah.edu.hn; 2 Pharmaceutical Technology Research Group, Faculty of Chemical Sciences and Pharmacy, National Autonomous University of Honduras (UNAH), Tegucigalpa 11101, Honduras; 3 Departamento de Química, Facultad de Ciencias Exactas y Naturales, Universidad Técnica Particular de Loja, San Cayetano Alto, Loja 1101608, Ecuadorxvjaramillo@utpl.edu.ec (X.J.-F.); 4 Institut de Nanociència i Nanotecnologia, Universitat de Barcelona (UB), Av. Diagonal 645, 27-31, 08028 Barcelona, Spain; silvadeabreu@ub.edu (M.S.-A.);; 5 Departament de Farmàcia i Tecnologia Farmacèutica, i Fisicoquímica, Facultat de Farmàcia i Ciències de l’Alimentació, Universitat de Barcelona (UB), Av. Diagonal 645, 27-31, 08028 Barcelona, Spain; 6 Laboratory of Parasitology, Department of Biology, Health, and Environment, Faculty of Pharmacy and Food Sciences, University of Barcelona (UB), Av. Diagonal 645, 27-31, 08028 Barcelona, Spain

**Keywords:** cutaneous leishmaniasis, amphotericin B, Pluronic^®^ F127, topical delivery, permeation studies, in vitro cytotoxicity, in vitro leishmanicidal activity

## Abstract

**Background/Objectives**: Leishmaniasis is a neglected tropical disease caused by a protozoan parasite of *Leishmania*. This study aimed to evaluate the in vitro efficacy and toxicity of a previously developed amphotericin gel as a possible treatment for cutaneous leishmaniasis. **Methods**: First, quality control of the AmB-gel was carried out, including microbiological stability. The permeated and retained drug was tested on healthy and lacerated human skin. Tolerance to the AmB-gel was tested in vitro using HaCaT, RAW 264.7, and J774 cell lines and by an irritation test (HET-CAM). Promastigotes and amastigotes of various *Leishmania* species were tested, and the microscopic morphology of promastigotes exposed to the formulation was analyzed. Computational analysis was performed on the drug, polymer, and ergosterol in the promastigote. **Results**: The AmB-gel presented appropriate characteristics for topical use, including no microbial contamination after storage. The amount of drug retained on the intact and injured skin was 1180.00 ± 13.54 µg/g/cm^2^ and 750.18 ± 5.43 µg/g/cm^2^, respectively. The AmB-gel did not cause significant signs of toxicity. The IC_50_ of the AmB-gel for promastigotes was less than 1 µg/mL for the four species examined, i.e., *Leishmania infantum*, *Leishmania tropica*, *Leishmania major*, and *Leishmania braziliensis*, and less than 2 µg/mL for amastigotes of *Leishmania infantum* and *Leishmania tropica*. The AmB-gel caused notable effects on the surface of promastigotes. Computational analysis revealed primarily hydrophobic and van der Waals interactions between AmB and Pluronic^®^ F127 and ergosterol. **Conclusions**: Based on the drug retention content and IC_50_ values observed for both parasite stages, the AmB-gel may be a promising candidate for in vivo studies in patients with cutaneous leishmaniasis.

## 1. Introduction

Leishmaniasis is a neglected tropical disease (NTD) caused by a protozoan parasite of *Leishmania*. According to the World Health Organization (WHO), between 700,000 and one million new cases are estimated to arise annually, and this disease is endemic in approximately 99 countries [1,2]. In numerous countries, leishmaniasis is significantly underreported due to unrecognized cases and the non-mandatory nature of reporting [3]. The life cycle of leishmaniasis begins with a bite from an infected female phlebotomine sandfly that injects the infectious promastigotes into the bloodstream. These are then phagocytosed by macrophages and transformed into amastigotes. The amastigotes multiply within infected cells and spread to different tissues, depending partly on the *Leishmania* species involved [4]. There are three types of leishmaniasis: visceral leishmaniasis, which affects organs including the spleen, liver, and bone marrow; mucocutaneous leishmaniasis, an aggressive form of cutaneous leishmaniasis that primarily damages the mucous membranes of the nose, mouth, and throat; and cutaneous leishmaniasis, the most common form of this disease, which causes skin lesions that begin as small red papules (initially 5 to 10 mm) and may spread to several areas of the body [5].

The treatments used for cutaneous leishmaniasis are diverse, and the first-line therapies consist of antimony salts such as sodium stibogluconate (Pentostam^®^, GlaxoSmithKline, London, UK) or meglumine antimoniate (Glucantime^®^, Sanofi, Paris, France), applied intravenously, intramuscularly, or intralesionally. However, these drugs can cause adverse effects, including severe pain at the administration site, nausea, vomiting, pancreatitis, renal and hepatic failure, as well as cardiotoxicity [6]. There are other treatments available, such as oral miltefosine. However, this drug has demonstrated reproductive toxicity and is thus contraindicated for pregnant women and patients with gastrointestinal disorders. Additionally, its half-life of seven days increases the risk of developing miltefosine-resistant parasites [7]. Another therapeutic alternative is the intramuscular injection of paromomycin, which produces pain at the puncture site and ototoxicity. Pentamidine is another alternative, but its use is limited due to cardiac toxicity, diabetes mellitus, hypotension, and gastrointestinal side effects [8]. Azole families have also been used as a leishmaniasis treatment due to their effect on blocking the parasites’ ergosterol synthesis. Ketoconazole, itraconazole, and fluconazole have been used for cutaneous leishmaniasis with variable cure rates. However, they produce high hepatotoxicity, and dose adjustment is required. Topical liposomal azithromycin has also been shown to have an effect against cutaneous leishmaniasis in clinical studies, with results similar to those produced by meglumine antimoniate. However, more studies are needed to demonstrate the efficacy of this drug [9,10].

Amphotericin B (AmB) is the second-line drug treatment for leishmaniasis. It works by binding to ergosterol in the cell membrane of the parasites, creating pores that ultimately lead to their death [11]. Currently, several types of AmB are on the market, such as AmB deoxycholate, which has been used for many years to treat systemic and complicated mycoses. This type of AmB has serious adverse effects, such as renal toxicity. For this reason, pharmaceutical industries have developed lipid formulations or colloidal suspensions to improve bioavailability and decrease the toxicity of this drug, e.g., AmBisome^®^ (Gilead Science, Foster City, CA, USA), Abelcet^®^ (McKesson, New York, NY, USA) and Amphocil^®^ (PENN PHARMACEUTICALS LIMITED, Cardiff, UK). However, these formulations are administered intravenously and are expensive, making them difficult to access in countries where leishmaniasis is endemic [12].

Due to several drawbacks, including the high cost of current drugs, their potential toxic effects, and the development of parasite resistance, new formulations must be developed to combat leishmaniasis [13]. A suitable topical formulation could be an alternative to attack *Leishmania* parasites present in the dermal layers of the skin. This type of administration has several advantages. These include ease of administration, good clinical response in non-extensive cutaneous and mucosal infections, rarity of side effects after use, no interference with the use of other oral or parenteral drugs, avoidance of adverse effects and drug interactions, no need for patient monitoring with analytical tests, and good cost-effectiveness in specific clinical situations [14]. Topical formulations can be designed to moisturize or enhance the penetration of an active ingredient, often a drug, into or through the skin. It is, therefore, important to deliver the drug in a biodegradable, biocompatible, and non-toxic excipient [15]. Based on this and the fact that there are no AmB-containing formulations on the market for dermal application, an AmB-gel was previously developed using Pluronic^®^ F127 (Fagron, Barcelona, Spain), an amphiphilic thermoreversible copolymer consisting of a central hydrophobic block of polypropylene oxide flanked by hydrophilic polyethylene blocks (PEG–PPO–PEG) [16]. This excipient is of interest in the design of dermal and transdermal administration to favor, improve, or delay the penetration of drugs through the skin, with healing properties characterized by its thermoreversibility, being liquid at a temperature of 2–8 °C and solid at 25 °C [17]. The main objective of this research was to evaluate the efficacy and toxicity of the AmB-gel for dermal application in cases of cutaneous leishmaniasis. The findings of this study, based on in vitro assays and an irritation test (HET-CAM), suggest that this formulation is safe for dermal application. Moreover, its in vitro efficacy was demonstrated in four species of promastigotes of *Leishmania infantum*, *Leishmania tropica*, *Leishmania major*, and *Leishmania braziliensis*, showing IC_50_ values less than 1 µg/mL and less than 2 µg/mL for amastigotes of *Leishmania infantum* and *Leishmania tropica*.

## 2. Results

### 2.1. Preparation and Characterization of the AmB-Gel

**Figure 1:**
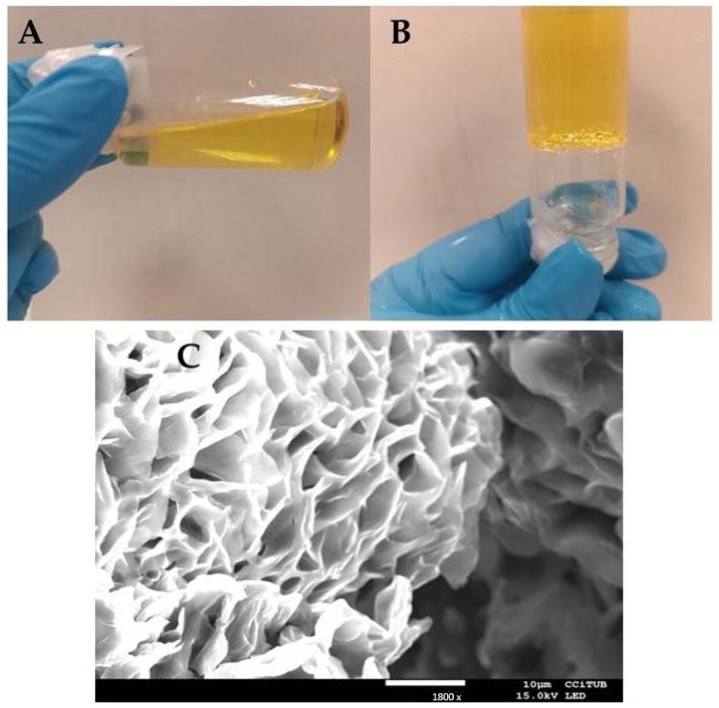
Characterization of AmB-gel. (**A**) AmB-gel at 4 °C; (**B**) AmB-gel at 32 °C; and (**C**) scanning electron microscopy (SEM) image of AmB-gel. Magnification 1800×.

The composition formula of the AmB gel (0.03%) consisted of 4.76% of DMSO, 23.8% of Pluronic^®^ F127, and 71.41% of water [16]. This formulation had a homogeneous appearance, translucent yellow color, pH between 5 and 6, and a porous microscopic structure, which is often found in the internal structure of gels made from Pluronic^®^ F127 (Figure 1). Usually, it looks like a sea sponge, with the drug incorporated into its pores and subsequently released [16].

The AmB-gel was liquid at low temperatures and stored in the refrigerator, showing a viscosity of 0.09 Pa·s and a Newtonian behavior at 4 °C. However, due to the negative thermoreversibility of the Pluronic^®^ F127, it became a relatively solid and sticky gel at temperatures above 25 °C, showing a viscosity of 3.75 Pa·s and a pseudoplastic behavior at 32 °C.

**Figure 2:**
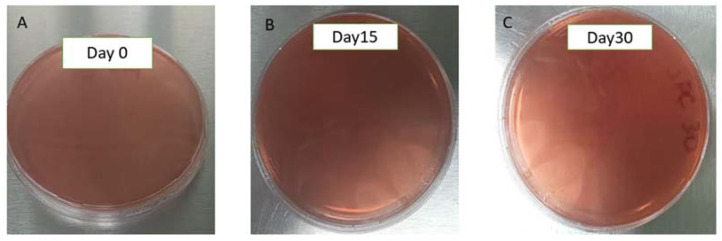
Microbiological control of AmB-gel. No growth of *Staphylococcus aureus* and *Pseudomonas aeuroginosa.* (**A**) AmB-gel culture on the day of preparation. (**B**) AmB-gel culture stored for 15 days. (**C**) AmB-gel culture stored for 30 days. At all storage temperatures, the AmB-gel did not show bacterial growth.

Microbiological characterization of the AmB-gel and blank gel confirmed the absence of microorganisms (<10 CFU/mL), including fungi and yeasts, as well as mesophilic aerobic microorganisms and Gram-negative microorganisms (total coliforms) under any of the conditions tested at 6 °C, 30 °C, and 40 °C for 0, 15, and 30 days. This result satisfies the requirements established for topical pharmaceutical products. In the same way, there was an absence of growth of *Staphylococcus aureus* or *Pseudomonas aeruginosa* on blood culture plates (Figure 2).

### 2.2. Ex Vivo Permeation Studies

No drug was found in the receptor compartment, demonstrating that AmB would not pass through the deep layers of healthy or injured skin. However, amounts of 1180.00 ± 13.54 µg/g/cm^2^ and 750.18 ± 5.43 µg/g/cm^2^ were retained on healthy and injured skin, respectively (Table 1).

### 2.3. Tolerance Studies

#### 2.3.1. In Vitro Cytotoxicity Assay

**Figure 3:**
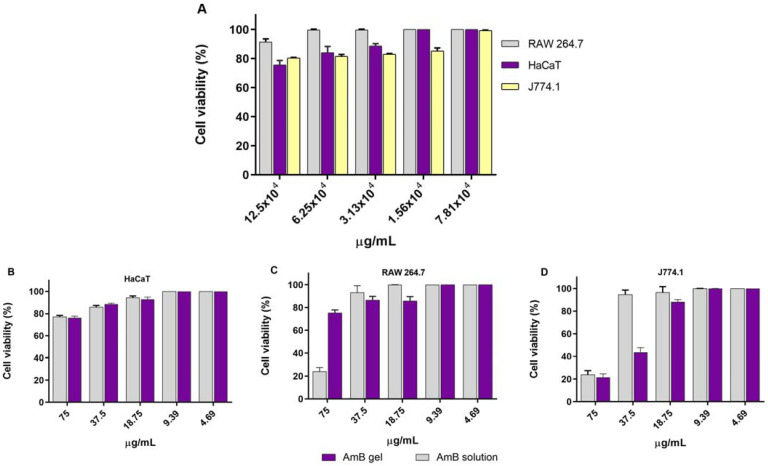
Cytotoxic effect in different cell lines. (**A**) Cytotoxicity of blank gel; (**B**) cytotoxicity of AmB-gel and AmB solution in HaCaT keratinocytes; (**C**) cytotoxicity of AmB-gel and AmB solution in RAW 264.7 macrophages; and (**D**) cytotoxicity of AmB-gel and AmB solution in J774.1 macrophages.

Although the solution and the gel showed a similar toxicity against the J774.1 cell line at 75.00 µg/mL, the AmB-gel showed a more significant toxicity against the RAW 264.7 cell line at 37.5 µg/mL. However, no toxicity signal was observed when the concentration was decreased for both macrophage lines. The AmB-gel had no cytotoxic effect on keratinocytes (HaCaT). Finally, the blank gel did not show toxicity against any cell line (Figure 3).

#### 2.3.2. Irritation Study: Hen’s Egg Test on the Chorioallantoic Membrane (HET-CAM)

**Figure 4:**
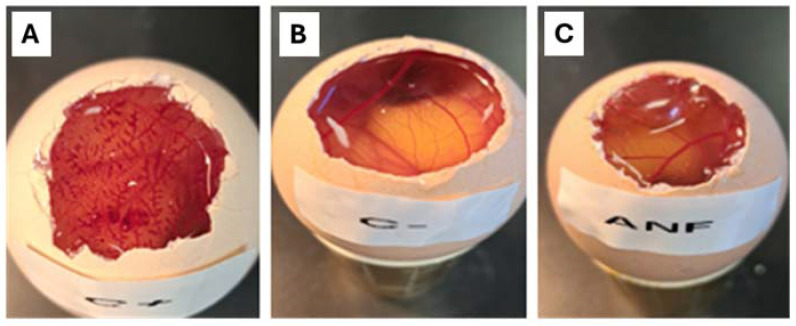
Irritation study: hen’s egg test on the chorioallantoic membrane (HET-CAM). (**A**) Positive control, (**B**) negative control, and (**C**) AmB-gel. All the OII values were calculated after 5 min of AmB-gel and solution application.

Figure 4C shows that after 5 min of applying the AmB-gel, a signal of irritation to the chorioallantoic membrane was detected, showing a value for the ocular irritation index (OII) below 0.9, classifying it as a non-irritant. However, the positive control showed a severe hemorrhage, categorizing the NaOH 0.1 N solution as an irritant. This can be observed in Figure 4A. In contrast, Figure 4B shows the negative control (NaCl 0.9%) did not produce any injury.

### 2.4. Efficacy Studies

#### 2.4.1. In Vitro Leishmanicidal Activity in Promastigotes and Amastigotes

**Figure 5:**
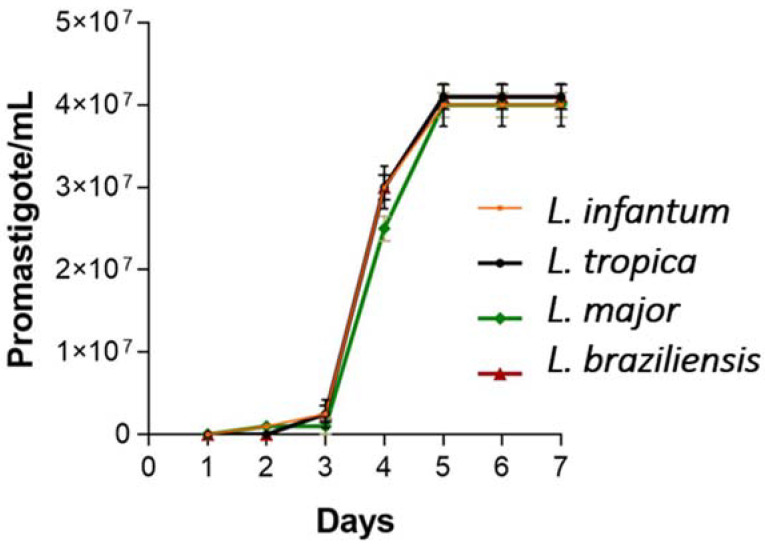
Exponential growth of the different *Leishmania* species studied.

The growth of promastigotes was observed and quantified in all *Leishmania* species studied. Exponential growth was observed on the seventh day of the experiment (Figure 5). In this sense, the *Leishmania* cultures from day 7 were used to carry out the activity tests regarding the AmB-gel formulation.

Table 2 summarizes the activity against promastigotes and amastigotes. The selectivity index (SI) values indicate the formulation’s ability to generate an antiparasitic effect against *Leishmania infantum* and *Leishmania tropica* amastigotes. The higher the SI value, the more significant the impact against the parasite. Infection was not achieved with *Leishmania major* and *Leishmania braziliensis* promastigotes in macrophages. The infection rates for these *Leishmania* species were approximately 20% and 25%, respectively.

#### 2.4.2. Scanning Electron Microscopy (SEM) for Promastigotes

**Figure 6:**
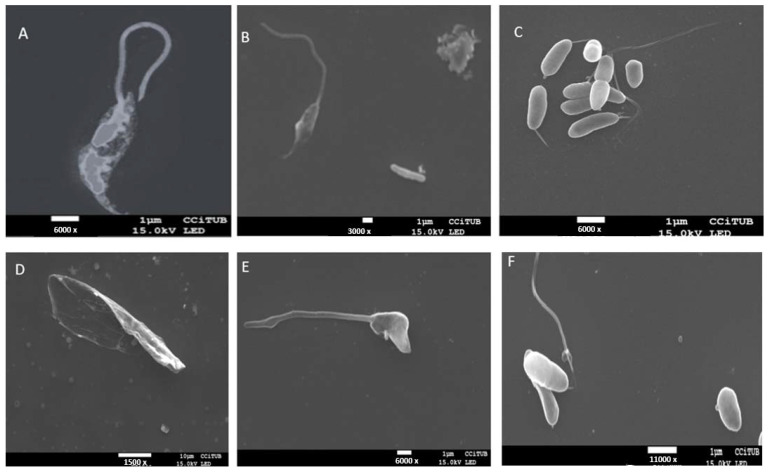
Images of promastigotes were obtained by scanning electron microscopy (SEM). (**A**) Promastigote without treatment. (**B**) Promastigote was exposed to blank gel; (**C**–**F**) Promastigote was exposed to AmB-gel.

Image 6 shows the promastigote with the head elongated as expected; no treatment was applied in this case. On the other hand, in Figure 6B, the promastigote is seen only with the excipient (blank gel). No structural changes are observed; the head is always elongated and attached to the flagellum. In Figure 6C, several promastigotes are observed with a structural change in the head (rounded) and some without their flagellum. Figure 6D shows that the promastigote only has the head and has lost the flagellum. In Figure 6E, one can also see the effect on the promastigote’s head. Finally, in Figure 6F, the head of the promastigote is rounded and divided in half, probably due to the effect of AmB released into the parasite. In these cases (Figure 6C–F), *Leishmania infantum* promastigotes were treated with the AmB-gel.

### 2.5. Computational Methods

**Figure 7:**
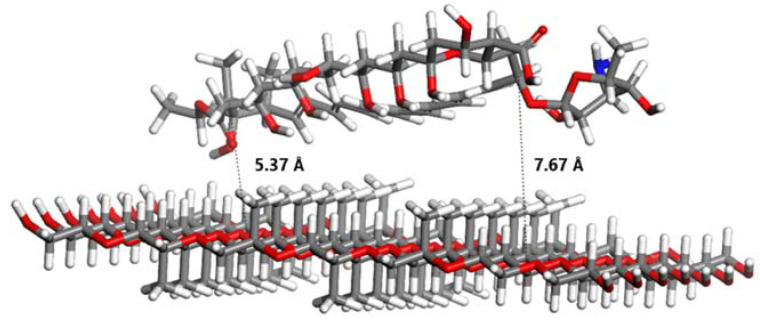
AmB-PF127 molecular interaction. Red bars indicate oxygen atoms, gray bars represent carbon atoms, white bars correspond to hydrogen atoms, and blue bars denote nitrogen atoms.

As shown in Figure 7, the specific atoms of Pluronic^®^ F127 that interact with AmB include the carbons of the methyl and methylene groups of poly (propylene oxide) (PPO), which form hydrophobic interactions with the aliphatic carbons and double bonds of the AmB ring. On average, the calculated distance between AmB and Pluronic^®^ F127 was 6.52 Å. In this study, the interaction energy between AmB and the hydrophobic core of Pluronic^®^ F127 was determined to be 3.86 kJ/mol.

**Figure 8:**
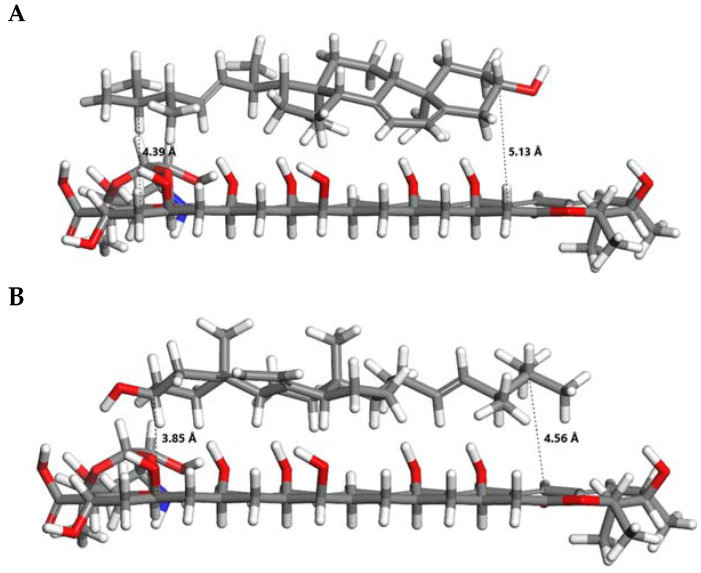
AmB–ergosterol molecular interaction. (**A**) Parallel orientation and (**B**) antiparallel orientation. Red bars indicate oxygen atoms, gray bars represent carbon atoms, white bars correspond to hydrogen atoms, and blue bars denote nitrogen atoms.

Regarding AmB–ergosterol interactions, the computational calculations provided the distances for each of the orientations. From Figure 8, the average distances obtained were 4.2 Å and 4.8 Å for the parallel and antiparallel orientations, respectively. On the other hand, the interaction energies between AmB and ergosterol were determined to be 7.38 kJ/mol and 7.07 kJ/mol for the parallel and antiparallel conformers, respectively. These findings align with those reported by other authors and reveal a consistent interaction mechanism between the hydrophobic polyene region of AmB and the sterol nucleus of ergosterol [18,19].

## 3. Discussion

Cutaneous leishmaniasis affects any body part, i.e., the face, arms, trunk, and legs, and can spread to mucous membranes such as the nose, ear, and eyes. It is considered a neglected disease and affects people with conditions of malnutrition and immunosuppression, but it is very common to find it in endemic countries. It is vector-borne, although some authors have also considered it a zoonosis [20,21]. The importance of treatment lies in developing an easy, fast, and economical formulation that is biocompatible with the skin and mucous membranes and less toxic than the available formulations [22]. In this study, an AmB-gel formulation has been proposed as a topical alternative for treating cutaneous leishmaniasis. It is a thermoreversible formulation with a liquid consistency, viscosity, and Newtonian behavior at low temperatures. However, when in contact with the skin, the gel formulation generates a depot effect with higher viscosity and pseudoplastic behavior. In cases of leishmaniasis, especially ulcerated ones, over-contamination should be avoided, so the application of medications in the area infected by *Leishmania* must be carried out carefully. Considering this approach, microbiological control of the gel was carried out, confirming that this formulation did not show the growth of microorganisms. Studies were carried out, so the risk of over-contamination is practically zero; however, to avoid contact with the skin, the gel could be supplied in a spray device, taking advantage of its low viscosity at low temperatures.

Ex vivo permeation studies provide valuable information to predict the in vivo behaviour of new formulations. Franz diffusion cells are widely used to determine drug permeation through the skin. The skin permeation study through human skin is considered the gold standard for evaluating drug delivery from a transdermal system [23]. In this study, lacerated human skin was used for the tests to simulate the skin in leishmaniasis processes where ulcerated cases occur. This experiment showed that AmB cannot penetrate the deep layers of the skin, as no drug was found in the receptor compartment. Previous studies have shown that AmB does not tend to permeate the skin [16,24,25,26]. This can be explained because AmB has a high molecular weight (926 Da), high hydrophobicity, and liposolubility, which would limit the passage of the drug through the dermis due to its aqueous structure. However, AmB could penetrate the stratum corneum and remain within the skin since a significant amount of the drug was extracted from the tissue at the end of the ex vivo permeation experiment. This suggests that the drug will not reach the systemic circulatory system, providing only a local effect in the target area without side effects, which is beneficial in the topical treatment of ulcers present in cutaneous leishmaniasis. In addition, it is important to note that in leishmaniasis processes, AmB could be retained in nodules and papules, exerting an eminently local effect [27]. Previous studies have shown that AmB can pass through rat skin in small amounts, but it is important to consider that rat skin is more permeable than human skin [27,28,29,30]. It has been shown that there are variations in skin based on age, sex, and breed, between animals or within the same animal, that could potentially affect the permeability of the substances to be tested [31]. Table 1 shows lower amounts of AmB were retained in the lacerated skin compared to healthy skin. This can be explained because the drug is highly lipophilic, and therefore, its retention is favored by the lipids present in the intact stratum corneum of healthy skin compared to the underlying layers of ulcerated skin. Similar results have been found in previous studies, which have confirmed greater affinity of AmB to the stratum corneum, suggesting that this layer of the skin acts as a reservoir for the drug. This local depot effect could prolong the pharmacological effect’s duration in treating nodules or papules of cutaneous leishmaniasis, which are covered by an epidermal layer [16,25,32].

Cell toxicity studies are essential, indicating whether a formulation can be administered. The AmB solution showed similar toxicity in both macrophage cell lines. However, the gel showed more significant toxicity in J774A cells than in RAW 264.7 cells. This latter cell line is known to exhibit multiple drug resistance properties caused by the presence of P-glycoprotein in the structure of these cells [33]. Blank gel was not toxic, indicating its safe use on the skin. This Pluronic^®^ F127-based gel meets all the requirements for in vitro and in vivo therapeutic and biomedical applications, such as biocompatibility, biodegradability, and low toxicity [25,34,35,36]. Cutaneous leshmaniasis affects the skin and mucous membranes. Skin sores usually start at the site of the sand fly bite. Some people may see sores on the mucous membranes, such as the nose and gums. Ulcers may also appear on the forehead, cheeks, and eye areas. Irritation testing on chicken embryos demonstrated that AmB-gel would not irritate the eyes or nasal mucosa if this formula were applied near these structures. The irritation test on chicken embryos showed that AmB-gel does not irritate the eyes or nasal mucosa if this formula is applied close to these structures. 

To carry out the leishmanicidal activity studies, the appropriate day for promastigotes of Leishmania to be in a stationary phase and be ready for axenic amastigote production was determined. Four species of *Leishmania* were used, including *Leishmania infantum* and *Leishmania tropica*, which usually cause cutaneous leishmaniasis. In the case of *Leishmania major*, it can also cause cutaneous leishmaniasis in humans and animals, and *Leishmania braziliensis* produces leishmaniasis that is more complicated to treat, such as mucocutaneous leishmaniasis [37]. Moreover, of the four species studied, *Leishmania tropica* is characterized as a species that causes recurrent leishmaniasis and is usually more resistant to drugs [38]. The IC_50_ values of the AmB-gel in this study for the four species of promastigotes studied were lower than 1 µg/mL, i.e., lower than those found for the AmB solution, so the formulation would be considered more effective than the solution. In the case of *Leishmania infantum* and *Leishmania major* amastigotes, the IC_50_ values were also lower for the AmB solution. The blank gel does not produce an antiparasitic effect. However, since it is not toxic, it is considered a suitable vehicle, in addition to the fact that the reparative effect of Pluronic^®^ F127 has been observed in lacerated skin, helpful in ulcerated-type cutaneous leishmaniasis processes. These findings have been previously demonstrated in rabbit models with lacerated skin whose histological analyses showed significant improvements in the healing processes of blank gels based on Pluronic^®^ F127. Likewise, it has a depot effect, which allows it to retain the AmB at the site of action for a more local effect [16]. In previous studies, the efficacy of various formulations made from AmB has been demonstrated in a range of IC_50_ values between 0.2 and 1.62 µg/mL, values similar to those found in this study (Table 2) [39,40]. However, the values vary since they depend on the infecting species and the excipients used in the formulation [41,42,43]. In addition, this study was carried out using SEM analysis to observe the promastigote morphology after being exposed to the formulation. Figure 6 shows the typical promastigote shapes with an elongated cell body in the control. However, cells treated with the AmB-gel formulation showed changes in the shape of the cell body that began to assume a rounded morphology and involvement of the flagellum. However, it was impossible to observe the presence of cellular debris or more damage to the cell membrane, which could imply cell death due to apoptosis [44,45].

**Figure 9:**
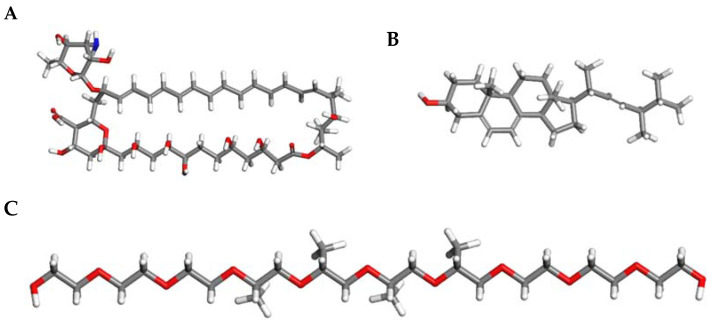
Molecular structure of (**A**) amphoterecin B; (**B**) ergosterol, and (**C**) Pluronic^®^ F127. Red bars indicate oxygen atoms, gray bars represent carbon atoms, white bars correspond to hydrogen atoms, and blue bars denote nitrogen atoms.

Amphotericin B (AmB) has a macrocyclic ring with hydrophobic regions formed by aliphatic carbons and conjugated double bonds. These features allow the macrocyclic ring of AmB to interact with nonpolar molecules through hydrophobic interactions. The carbon and hydrogen atoms in the ring participate in these interactions, which are primarily van der Waals forces. As shown in Figure 9, AmB also has a hydrophilic face with hydroxyl (-OH) groups that tend to interact with polar molecules or water. The interaction between amphotericin B (AmB) and Pluronic^®^ F127 in pharmaceutical formulations involves complex molecular mechanisms that enhance drug delivery and reduce toxicity. Pluronic^®^ F127, a triblock copolymer, is utilized to form micelles that encapsulate drugs, improving their solubility and bioavailability. Combining these two components in drug formulations leverages the unique properties of each to optimize therapeutic outcomes. Pluronic^®^ F127 contains a hydrophobic core made of poly(propylene oxide) (PPO), whose structure consists of repeating propylene oxide units (-CH(CH_3_)-O-CH_2_-). In this core, hydrophobic interactions are established between the carbon atoms of the methyl (CH_3_) and methylene (CH_2_) groups of PPO and the carbon atoms of AmB’s macrocyclic ring. These interactions are based on van der Waals forces, which are weak but essential for stabilizing amphotericin on the hydrophobic surface of the Pluronic^®^ F127 copolymer. The calculated distance between AmB and Pluronic^®^ F127 was 6.52 Å. This interaction stabilizes AmB on the copolymeric surface, improving its solubility in aqueous solution and reducing its toxicity by preventing aggregate formation. In this study, the interaction energy between AmB and the hydrophobic core of Pluronic^®^ F127 was determined to be 3.86 kJ/mol. This value indicates the stability of amphotericin B within the hydrophobic core of Pluronic^®^ F127, where van der Waals interactions play a key role in stabilizing the system, contributing to enhanced solubility and reduced drug toxicity. This computational estimate is an approximation and would depend on factors such as the exact geometry of the interaction and the system’s environment (temperature, solvent, etc.), but it provides a rough estimate of the magnitude of the energy involved in the adsorption of amphotericin B onto Pluronic^®^ F127.

Regarding AmB–ergosterol, the interaction occurred predominantly between the hydrophobic polyene region of AmB and the sterol nucleus of ergosterol. This type of interaction is consistent with hydrophobic interactions and van der Waals (VDW) forces, which are critical for stabilizing the AmB–ergosterol complex [46]. Additionally, the conjugated polyene chain of AmB and the planar tetracyclic core of ergosterol allow for potential π–π stacking interactions, further enhancing the affinity and stability of the complex. These interactions have significant implications for the mechanism by which AmB disrupts the cellular membrane of *Leishmania*. The stable binding of AmB to ergosterol facilitates the aggregation of AmB molecules into oligomers that form transmembrane pores. These pores disrupt ion homeostasis by allowing the uncontrolled passage of ions like K^+^, Na^+^, and H^+^, leading to osmotic stress and membrane depolarization [47]. In addition to pore formation, the strong affinity of AmB for ergosterol can lead to the sequestration of sterol from the membrane, further compromising its structural and functional integrity. This so-called “sterol sponge” effect destabilizes the lipid bilayer and diminishes the organization and function of membrane-associated proteins [48,49].

## 4. Materials and Methods

### 4.1. Materials

The AmB used for the study was obtained from Acofarma (Barcelona, Spain). Pluronic^®^ F127 excipient was obtained from Fagron (Barcelona, Spain). Dimethyl sulfoxide (DMSO), acetonitrile, and acetic acid were obtained from Sigma–Aldrich (Darmstadt, Germany). Transcutol^®^ P and propylene glycol were kindly provided by Gattefossé (Barcelona, Spain). The water used in all experiments was obtained from a Milli-Q^®^ Plus System (Millipore Co., Burlington, MA, USA). All the chemicals and reagents were of analytical grade.

### 4.2. Strains and Parasite Cultures

The *Leishmania* species used for the trials were isolated from patients with cutaneous leishmaniasis in Barcelona, Spain. The samples were coded as follows: *Leishmania tropica* (MHOM/ES/2010/BCN-809), *Leishmania infatum* (MHOM/ES/2014/BCN-855), *Leishmania major* (MHOM/ES/2011/BCN-839), and *Leishmania braziliensis* (MHOM/BR/88 BCN-25).

### 4.3. Preparation and Characterization of the AmB-Gel 

The AmB-gel was prepared by dissolving the drug in DMSO under magnetic stirring and incorporating this drug solution into a base gel prepared with Pluronic^®^ F127 completely dissolved in cold water [16].

The pH value of the obtained formulation was determined using a Crison 501 digital pH/mV-meter (Crison Instruments, Barcelona, Spain).

The internal structure of the AmB-gel was evaluated using a previously dried sample of formulation coated with carbon as a conductor agent, visualized under a scanning electron microscope using a JEOL J-7100F (Peabody, MA, USA).

The rheology of samples of the AmB-gel at 4 and 32 °C was examined using a Thermo Scientific Haake Rheostress 1 rotational rheometer (Thermo Fisher Scientific, Kalsruhe, Germany) [16].

A microbiological control of the developed formulation was carried out to ensure that it is suitable for application in areas ulcerated by the disease. To this end, a 1:10 dilution of the AmB-gel and the drug-free gel (blank gel) was placed in peptonized water until three concentrations were obtained: 10^−1^, 10^−2^, and 10^−3^. Then, 100 μL of the dilutions was seeded in duplicate on plates with different culture media (n = 2). Three culture media were used: Sabouraud dextrose agar (SDA, for fungi and yeasts), SMA standard agar (standard method agar, for mesophilic aerobic microorganisms), and VRBA (neutral red–violet-crystal bile agar, for Gram-negative microorganisms). Likewise, different AmB-gel concentrations and blank gel concentrations were seeded on blood culture plates to observe the growth of *Staphylococcus aureus* and *Pseudomonas aeruginosa*. Subsequently, the SMA, VRBA, and blood gelose plates were incubated for 24 and 48 h at 37 ± 0.5 °C in a Thermo Scientific Heratherm incubator (Waltham, MA, USA), and the SDA plates were incubated for 5 days at 25 °C (room temperature). Microbiological analysis for the isolation of mesophilic aerobic microorganisms, total coliforms, molds, and yeasts was evaluated at 0, 15, and 30 days at 6 °C and in a monitored environment (30 °C and 40 °C), with a variation of ±2 °C. After incubation, colonies were counted to calculate colony-forming units per milliliter or gram of product. Growth of *Staphylococcus aureus* and *Pseudomonas aeruginosa* should be absent.

### 4.4. Ex Vivo Permeation Studies

AmB-gel permeation tests were carried out using Franz diffusion cells using intact human skin and injured human skin as membranes, the latter simulating the processes of ulcerated leishmania. The skin used was obtained from a donor undergoing plastic surgery at the SCIAS Hospital of Barcelona after written informed consent by the Ethical Committee of the Hospital of Barcelona (protocol N°002; dated 17 January 2020). Human skin was cut with a Zimmer^®^ dermatome (Zimmer biomet, Varsovia, IN, USA) into 400 µm thick samples, and its integrity was verified by measuring transepidermal water loss (TEWL) parameters. To injure the human skin tissue, the stratum corneum of healthy skin was partially removed by applying an adhesive tape 7 times. Transcutol^®^ P (Gattefossé, Barcelona, Spain) was used as receptor medium at 32 ± 0.5 °C with constant agitation. A 0.15 g of AmB-gel (0.03%) sample was deposited in the donor compartment using a diffusion area of 0.64 cm^2^. To extract the drug retained in the skin, pieces of skin were removed from Franz diffusion cells, and they were cut, weighed, and washed with a 0.05% sodium lauryl sulfate solution and subsequently with distilled water. The extraction was carried out by puncturing the skin pieces with a needle, and after weighing the skin pieces, they were added to 1 mL of Transcutol^®^ P and placed in an ultrasound bath for 30 min.

The determination of the amount of AmB from the permeation samples and that extracted from the skin pieces was analyzed using the HPLC method previously validated and described by Sosa et al. (2017) [24]. AmB was determined using a Waters^®^ 515 HPLC Pump, a 717 Plus Autosampler, and a 2487 Dual Absorbance Detector (Waters^®^, Milford, MA, USA). The assay used a Kromasil^®^ Eternity C18 column (250 mm × 4.6 mm × 5 µm, Teknokroma, Barcelona, Spain). The mobile phase was a mixture of acetonitrile, acetic acid, and water (52:4.3:43.7 *v*/*v*/*v*) and was pumped through the C18 column at a flow rate of 0.5 mL/min. A volume of 10 µL was injected per sample, and finally, the elute was analyzed at 406 nm. All measurements were performed at RT under isocratic conditions of elution. The calibration curves were prepared with freshly prepared stock solutions of AmB ranging from 0.39 to 200 µg/mL. The analytical method was precise, with a coefficient of variation between 0.02% and 8.79%, a relative percent error between −1.16% and 3.46%, and linear within the range of concentrations used (0.39–200 µg/mL), with a *p*-value of 0.05 corresponding to the ANOVA applied to the mean values [19].

### 4.5. Tolerance Studies

#### 4.5.1. In Vitro Cytotoxicity Test

RAW 264.7 and J774A.1 macrophage cell lines as well as a HaCat keratinocyte cell line (Eppelheim, Germany) were used to verify the cell toxicity of the AmB-gel. Previously, a suspension of 5.0 × 10^4^ cells/mL of each cell line was seeded in 96-well plates (Costar 3596, Corning Incorporated, Corning, NY, USA) and incubated at 37 °C and 5% CO_2_ atmosphere in RPMI-1640 complete medium supplemented with 10% heat-inactivated fetal bovine serum and 1% penicillin (100 U/mL)–streptomycin (100 mg/mL) solution (100 mg/mL) for 24 h. Serial double dilutions of the AmB-gel, blank gel, and AmB solution dissolved in DMSO were added and incubated for another 24 h. Finally, 10% WST-1 reagent (Roche Diagnostics GmbH, Paris, France) was added to all wells and incubated for 4 h under the same temperature conditions and CO_2_ atmosphere. Absorbance was read at 450 nm (Multiskan EX, ThermoElectron Corporation, Shanghai, China). To determine the concentration inhibiting 50% of cell viability (CC_50_), linear regression analysis was performed. The experiment was performed in triplicate [20].

#### 4.5.2. In Vitro Hen’s Egg Test on the Chorioallantoic Membrane (HET-CAM)

The irritation HET-CAM test was assessed to ensure that the formulation of the AmB-gel was non-irritating when administered as eye drops. First, 300 μL of the formulation studied was applied to the chorioallantois membrane of a fertilized chicken egg and monitored for 5 min after application. The following phenomena were considered: irritation, coagulation, and hemorrhaging. The test was developed using 3 eggs for each group (AmB-gel, negative control (0.9% NaCl), positive control (NaOH 0.1 N)). The ocular irritation index (OII) was calculated as the sum of the scores of each injury according to the following expression (Equation (1)):

(1) $$\frac{\left(301-H\right)\times 5}{300}+\frac{\left(300-V\right)\times 7}{300}+\frac{(300-C)\times 9}{300}$$

where *H*, *V*, and *C* are times (in seconds) until the start of the hemorrhage (*H*), vasoconstriction (*V*), and coagulation (*C*), respectively. The formulations were classified according to the following scores: OII ≤ 0.9 non-irritating; 0.9 < OII ≤ 4.9 weakly irritating; 4.9 < OII ≤ 8.9 moderately irritating; 8.9 < OII ≤ 21 irritating.

### 4.6. Efficacy Studies

#### 4.6.1. In Vitro Leishmanicidal Activity in Promastigotes

Promastigote growth curves were performed by counting them daily for 6–7 days to determine their kinetics and establish the logarithmic phases of growth since drug sensitivity tests are performed in this phase. The promastigotes obtained were grown at 26 °C in Schneider’s culture medium at pH 7.0 supplemented with 20% heat-inactivated fetal bovine serum, 25 µg/mL gentamicin solution (Sigma, St. Louis, MO, USA), and 1% penicillin (100 U/mL)–streptomycin (100 mg/mL) solution (Sigma, St. Louis, MO, USA).

The antimicrobial activity of the AmB-gel, blank gel, and AmB solution was evaluated. For this purpose, serial two-fold dilutions of the AmB-gel and AmB solution were made using Schneider’s culture medium and placed in a 96-well plate (Costar 3596, Corning Incorporated, Corning, NY, USA). A suspension of 1 × 10^6^ promastigotes/mL (in log phase) was added to the previous dilutions and incubated at 26 °C for 48 h. Parasitic growth was evaluated using the acid phosphatase method [21]. Briefly, the samples were lysed, and the enzymatic reaction with *p*-nitrophenyl phosphate was carried out using alkalinization. Optical density was read at 405 nm (Multiskan EX, ThermoElectron Corporation, Shanghai, China). The IC_50_ (the concentration inhibiting 50% of parasite growth) was calculated using variable transformation and linear regression analysis. The experiments were performed in triplicate.

Once the well that contained the promastigotes with the formulation that yielded the IC_50_ was identified, these promastigotes were observed by scanning electron microscopy (SEM). These samples were collected in Eppendorf tubes with a capacity of 1 mL and centrifuged at 14,000 rpm for 15 min at room temperature. The supernatant was removed, leaving the pellet at the bottom of the container. The pellets were washed three times for 10 min with 0.1 M phosphate buffer at pH 7.2. In the last wash, the pellets were resuspended in the same solution. This promastigote solution was deposited on a glass coverslip coated with poly L lysine to fix the parasites with a 2.5% (*v*/*v*) glutaraldehyde solution for 2 h. After this time, the samples were washed three times for 10 min with a 0.1 M phosphate solution at pH 7.2. The promastigotes were washed twice for 10 min with ethanol (30, 70, 75, 90, and 100%). Each sample was stored in 100% ethanol at 5 °C for 24 h to find the critical point. Finally, the samples were covered with carbon. The images were observed using scanning electron microscopy (SEM) using a JEOL J-7100F device (JEOL Peabody, Dearborn, MI, USA).

#### 4.6.2. In Vitro Leishmanicidal Activity in Amastigotes

The RAW 264.7 cell line was used to study the activity of the AmB-gel, blank gel, and AmB solution against amastigotes. A concentration of 5 × 10^4^ cells/mL (in an 8-well chamber slide of the LabTek system Nunc^®^, Rochester, NY, USA) was seeded and incubated for 24 h at 37 °C in a 5% CO_2_ atmosphere. Promastigotes of *Leishmania infantum*, *Leishmania major*, *Leishmania tropica*, and *Leishmania braziliensis* species were added to the cells in a 1:10 ratio (macrophages: parasites) and incubated for 24 h under the same conditions. After removing (by washing with sterile 0.1 M PBS) free promastigotes, fresh RPMI-1640 was added as fresh culture medium with serial 2-fold dilutions of gels and AmB in solution and incubated for 48 h under the same conditions. Untreated cultures were included as positive control. Slides were fixed and stained with Giemsa stain and counted to assess the number of amastigotes in 300 macrophages to determine the percentage of infected cells (in triplicate). The IC_50_ was expressed as the percentage growth inhibition relative to untreated controls [22].

### 4.7. Computational Methods

In addition to the experimental methods, computational methods were carried out to theoretically evaluate the chemical interaction between the gel polymer (Pluronic^®^ F127) and AmB as well as the chemical interaction between AmB and the ergosterol present in the promastigote. This computational study was carried out using density functional theory (DFT) with the Gaussian 16 software package (Gaussian, Inc., Wallingford, CT, USA). The initial atomic coordinates of AmB, Pluronic^®^ F127, and ergosterol were obtained from the open chemistry database PubChem. The B3LYP hybrid exchange-correlation function, which combines gradients from DFT and Hartree–Fock (HF) theory, was used to improve the precision in describing the electronic, geometric, and energetic interactions of the studied molecules. The TZVP basis set previously was employed to accurately represent molecular properties, balancing the description of valence orbitals with computational demand [50]. This basis set allowed for a detailed representation of the molecules’ electronic structure, ensuring reliable predictions of the stability and reactivity of the studied chemical species. A root-mean-square criterion for the density matrix convergence in the self-consistent field (SCF) iteration of 10^−14^ atomic units was established, with an energy convergence threshold of at least 10^−15^ atomic units, ensuring precision in determining the total energies of the molecular structures. Additionally, water was used as the solvent in all simulations, with its dielectric constant adjusted to 78.4, aligning the simulations with real experimental conditions due to the biological relevance of the solvent. Finally, the visualization of all molecular structures and properties was achieved using GaussView version 6 (Semichem Inc., Shawnee Mission, KS, USA), which provided a clear representation of the obtained results. Figure 9 shows the molecular structures used in this study.

All adsorption calculations were performed using the Adsorption Locator module within the BioVia Materials Studio, version 5.5 (BioVia, San Diego, CA, USA) software package. This module allows the simulation of pure and mixed adsorbates, with fixed composition, on a substrate. The Adsorption Locator module identifies potential adsorption sites by performing Monte Carlo searches of the configurational space of the substrate–adsorbate system as the temperature is slowly decreased within the simulated annealing process of a molecular dynamics run. In this work, we simulate the interactions between amphoterecin B and Pluronic^®^ F127 (AmB-PF127) as well as between amphoterecin B and ergosterol (AmB–erg). For the AmB–erg simulation, two orientations parallel (head-to-head) and antiparallel (head-to-tail) were considered to acquire the relative interaction strengths of the different orientations and to provide information on the molecular orientation preferences. The isosteric heat, or adsorption enthalpy, can be extracted from the adsorption calculation and is an average of all lowest energy configurations (obtained by Monte Carlo simulations) returned after each annealing cycle of the MD run. In total, 50 cycles containing 1,000,000 steps per cycle with annealing temperatures ranging from 298 to 500 K were used to obtain reproducible results. The Monte Carlo parameters were set according to the literature [1], a probability of 0.29 (ratio = 1) for ’conformer’, ’rotate’, and ’translate’ while ’regrow’ was set to 0.14 (ratio = 0.5). The COMPASS II force field was used together with atomic charges calculated using CASTEP single-point energy calculations. Ewald- and cluster-based summation methods were chosen for the electrostatic and van der Waals energy components, respectively, with the cutoff value set to 25 Å.

## 5. Conclusions

In conclusion, this study demonstrates that the topical application of an AmB-loaded Pluronic^®^ F127 gel to the affected area of cutaneous leishmaniasis has significant in vitro efficacy against promastigotes of four *Leishmania* species, i.e., *Leishmania infantum*, *Leishmania tropica*, *Leishmania major*, and *Leishmania braziliensis*, as well as amastigotes of *Leishmania infantum* and *Leishmania tropica* species while maintaining a favorable safety profile. The drug’s ability to remain within the skin suggests a localized therapeutic effect, which reduces the risk of adverse effects associated with known systemic treatments such as nephrotoxicity and hepatotoxicity. The thermoreversibility of the AmB-gel allows its easy application by a spray device, thus avoiding direct contact with infected skin and preventing the formulation from being infected. In addition, its microbiological stability guarantees safe use in ulcerated lesions without concomitant infectious diseases. Computational analysis revealed primarily hydrophobic and van der Waals interactions between AmB and Pluronic^®^ F127 and with the ergosterol of the parasite, ensuring the drug’s efficacy. Further studies, including the long-term stability of the formulation and in vivo validation, are needed to confirm its therapeutic efficacy and safety in clinical settings. However, the findings of this study lay the groundwork for future research and potential clinical applications, as well as for exploring combination therapies to improve treatment outcomes that provide alternatives to current therapies.

## Figures and Tables

**Table 1:** Amount of AmB permeated and retained on human skin.

	AmB Retained (µg/g/cm^2^)	% of Retention
Intact skin	1180.00 ± 13.54	67.13 ± 1.21
Injured skin	750.18 ± 5.43	42.68 ± 0.73

**Table 2:** In vitro leishmanicidal activity in promastigotes and amastigotes.

Strain	Compounds	IC_50_ (µg/mL)		CC_50_ RAW 264.7	SI
		Promastigote	Amastigote		
*L. infantum*	AmB-S	0.93 ± 0.25	1.50 ± 0.29	56.34 ± 0.39	37.56
	AmB-gel	0.62 ± 0.15	0.82 ± 0.34	81.61 ± 0.37	99.52
	Blank gel	>25,000	>25,000		
*L. tropica*	AmB-S	0.63 ± 0.05	1.79 ± 0.02	56.34 ± 0.39	31.47
	AmB-gel	0.37 ± 0.01	1.29 ± 0.01	81.61 ± 0.37	63.26
	Blank gel	>25,000	>25,000		
*L. major*	AmB-S	0.70 ± 0.01	NA	56.34 ± 0.39	NA
	AmB-gel	0.46 ± 0.30	NA	81.61 ± 0.37	NA
	Blank gel	>25,000	NA		
*L. braziliensis*	AmB-S	0.48 ± 0.01	NA	56.34 ± 0.39	NA
	AmB-gel	0.40 ± 0.06	NA	81.61 ± 0.37	NA
	Blank gel	>25,000	NA		

CC_50_: Cytotoxic concentration 50; IC_50_: inhibitory concentration in which 50% growth is inhibited compared to control growth; SI: selectivity index; RAW 264.7: macrophage cell line; NA: not applicable.

## Data Availability

Data presented in this study are contained in the article.
